# Emphysematous cholecystitis: A deadly disguise of a common culprit

**DOI:** 10.1002/ccr3.4341

**Published:** 2021-06-23

**Authors:** Ashesha Mechineni, Rajapriya Manickam, Balraj Singh

**Affiliations:** ^1^ St Joseph's University Medical Center Paterson NJ USA

**Keywords:** abdominal pain differential, acute cholecystitis, emphysematous cholecystitis

## Abstract

Emphysematous cholecystitis is a critical differential diagnosis for physicians to consider when evaluating patients with acute abdomen. Patients who are unable to undergo emergency surgery can benefit from percutaneous cholecystostomy.

## Graphical abstract

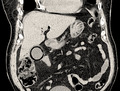

A 69‐year‐old male patient presents with a one‐day history of right upper quadrant pain and positive Murphy's sign on examination. Serology revealed leukocytosis (WBC 13.8*10e3/uL), normal liver panel (ALP 68 IU/L, AST 21 IU/L, ALT 19 IU/L). Past medical history was notable for hypertension, diabetes mellitus, coronary artery disease, chronic systolic heart failure, aortic valve replacement, paroxysmal atrial fibrillation, cerebrovascular accident, and chronic kidney disease stage 4. Ultrasonography showed mildly thickened gall bladder wall, no gall stones, or pericholecystic fluid. Computed abdominal tomography (CT) without contrast revealed emphysematous cholecystitis with typical findings of air in the gall bladder wall and biliary tree (Figures 1 and 2). The patient deemed high risk for surgery and underwent a percutaneous cholecystostomy. Patient proceeded to have laparoscopic cholecystectomy one month later with good clinical outcome.

**FIGURE 1:**
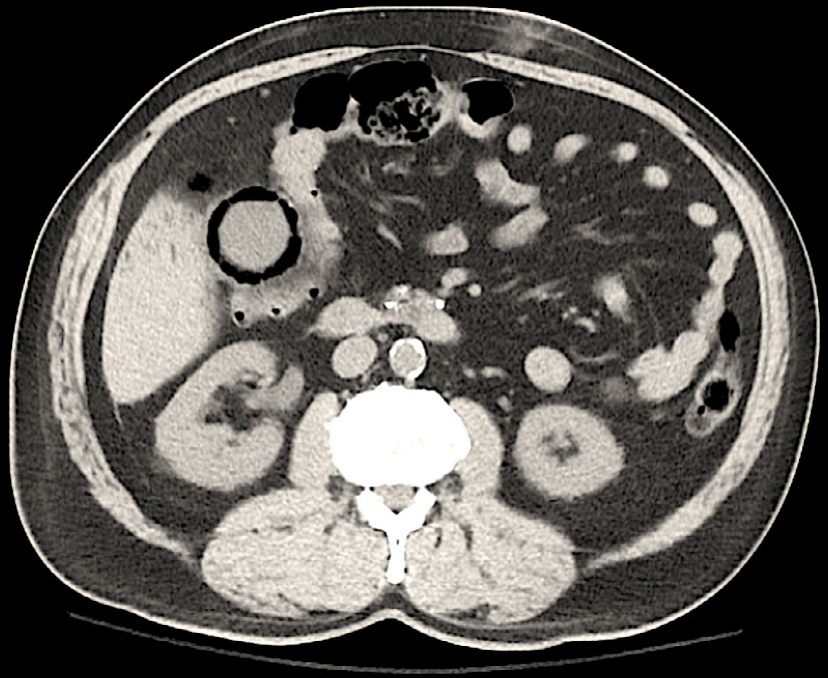
Cross‐sectional CT image of abdomen and pelvis showing air within gall bladder wall, a typical sign of emphysematous cholecystitis. Also noted are chronic calcific pancreatitis changes

**FIGURE 2:**
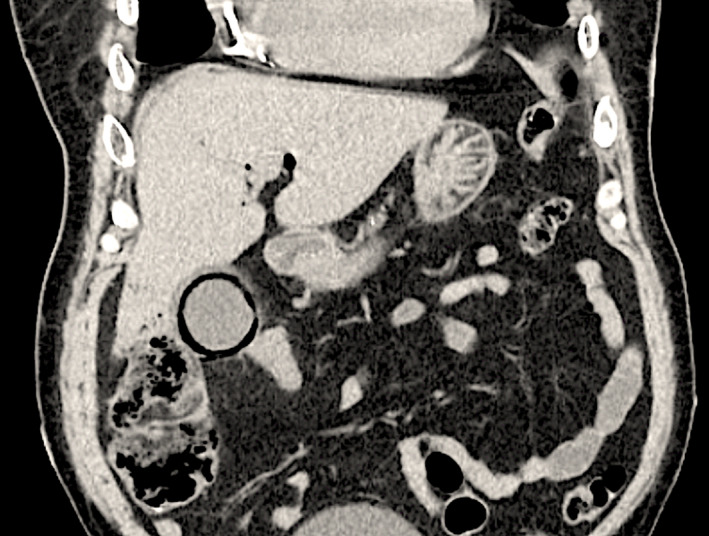
Coronal section view of CT abdomen showing thickening of the gall bladder wall surrounding emphysematous changes. Also seen are calcific pancreatitis and distended urinary bladder

Emphysematous cholecystitis is a form of acute cholecystitis with a high mortality rate (15%).^1^ The air in the gallbladder wall causes the pathognomonic features on imaging. The CT scan is the most sensitive and specific diagnostic test. Hepatic abscess, retroperitoneal air, and entero‐biliary fistula are important differential diagnoses. Although cholecystectomy is the gold standard of care, percutaneous cholecystostomy has shown to be effective in patients who are not surgical candidates.^2^

## 1 ETHICS STATEMENT

Patient consent has been obtained for this case report and is available for review upon request. Institutional Review Board, St. Joseph's University Medical Center policies were followed throughout the submission process.

## CONFLICT OF INTEREST

The authors declare no competing financial interests.

## AUTHOR CONTRIBUTIONS

A.M is responsible for the creation, drafting, and writing of manuscript including submission. B.S is responsible for image acquisition, editing, and writing of manuscript. R.M is responsible for final editing, proof reading, and revision of manuscript.

## STATEMENT ABOUT DIGITAL PHOTOGRAPHS

The authors declare that all digital photographs in this submission have not been edited, modified, or adulterated in any way.

## Data Availability

The data that support the findings of this study are available from the corresponding author, Ashesha Mechineni, MD, upon reasonable request.
